# Insecticide resistance and vector control.

**DOI:** 10.3201/eid0404.980410

**Published:** 1998

**Authors:** W G Brogdon, J C McAllister

**Affiliations:** Centers for Disease Control and Prevention, Atlanta, Georgia 30333, USA. wgb1@cdc.gov

## Abstract

Insecticide resistance has been a problem in all insect groups that serve as vectors of emerging diseases. Although mechanisms by which insecticides become less effective are similar across all vector taxa, each resistance problem is potentially unique and may involve a complex pattern of resistance foci. The main defense against resistance is close surveillance of the susceptibility of vector populations. We describe the mechanisms of insecticide resistance, as well as specific instances of resistance emergence worldwide, and discuss prospects for resistance management and priorities for detection and surveillance.


### Insecticide Resistance and Vector Control

**William G. Brogdon and Janet C. McAllister**
Centers for Disease Control and Prevention, Atlanta, Georgia, USA

Insecticide resistance has been a problem in all insect groups that serve as vectors of emerging diseases. Although mechanisms by which insecticides become less effective are similar across all vector taxa, each resistance problem is potentially unique and may involve a complex pattern of resistance foci. The main defense against resistance is close surveillance of the susceptibility of vector populations. We describe the mechanisms of insecticide resistance, as well as specific instances of resistance emergence worldwide, and discuss prospects for resistance management and priorities for detection and surveillance.

Many new and reemerging diseases are transmitted by arthropod vectors. Mosquitoes transmit malaria (1,2), dengue-dengue hemorrhagic fever (DHF) (3-5), yellow fever (6), Venezuelan equine encephalitis (3,7), and filariasis (8); sand flies transmit leishmaniasis (7); ticks transmit Lyme disease and ehrlichiosis (9,10); fleas and lice transmit *Bartonella* (11); and fleas, lice, and ticks transmit various rickettsioses (12-14). Resistance to insecticides has appeared in the major insect vectors from every genus. As of 1992, the list of insecticide-resistant vector species included 56 anopheline and 39 culicine mosquitoes, body lice, bedbugs, triatomids, eight species of fleas, and nine species of ticks (15). Other insects of public health importance, such as certain flies and cockroaches, show resistance in all genera.

Resistance has developed to every chemical class of insecticide, including microbial drugs and insect growth regulators. Despite decades of international efforts, a detailed practical description of insecticide resistance that would allow control strategies to be adjusted to specific needs remains the exception rather than the rule.

Insecticide resistance is expected to directly and profoundly affect the reemergence of vector-borne diseases (1), and where resistance has not contributed to disease emergence, it is expected to threaten disease control (15). However, careful scrutiny of current information about vector resistance (e.g., the World Health Organization [WHO] resistance database and records of control programs) shows that the full effect of resistance on control efforts is not known. Many instances of resistance reported for vector species and their regional or countrywide distribution are based on single datasets from a single point within a country and may be years, if not decades, old. Researching every resistance problem and every application of vector control is not practical. Yet control measures have to be selected for use, often at times of emergency. Although alternatives to vector control with insecticides are available, drug resistance problems (e.g., malaria) or vaccine cost and availability (e.g., Japanese encephalitis) make vector control an important option (1,16). Shrinking availability of insecticides as a result of resistance is exacerbated by removal from the market of insecticides no longer registered for public health use, especially in the past decade; the cost to keep certain compounds on the market is higher than can be recouped from such use. In addition, insecticide use is also monitored and restricted by regulatory agencies.

The potential of resistance to interfere with emergency use of insecticides first became apparent in 1993 when flooding in nine midwestern states increased the threat over the next 2 years of arboviral disease transmission (17). Most of the nine states affected had no public health entomologic or vector control

Address for correspondence: William G. Brogdon, Centers for Disease Control and Prevention, F22, 1600 Clifton Road, NE, Atlanta, GA 30333, USA; fax: 770-488-7794; e-mail: wgb1@cdc.gov.

resources, and none had susceptibility data for their vector mosquitoes. Preliminary data showed that resistance to the insecticides proposed for emergency use was widespread throughout the Midwest. As a result of these findings, a resistance surveillance laboratory was established at the Centers for Disease Control and Prevention (CDC), Atlanta, Georgia. Data collected by this laboratory in the last 3 years confirm that states vary enormously in their resources to deal with insecticide resistance. At present, 26 states participate in the Emerging Infectious Disease insecticide resistance surveillance project.

We provide an update on resistance of disease vectors to insecticides, use specific instances of emerging resistance to illustrate this complex, worldwide problem, and offer strategic priorities for combating it.

### Resistance Mechanisms

Insecticide resistance mechanisms (as opposed to insecticide avoidance behaviors important in the control of malaria vectors) have a biochemical basis (Figure 1). The two major forms of biochemical resistance are target-site resistance, which occurs when the insecticide no longer binds to its target, and detoxification enzyme-based resistance, which occurs when enhanced levels or modified activities of esterases, oxidases, or glutathione S-transferases (GST) prevent the insecticide from reaching its site of action. An additional mechanism based on thermal stress response has been proposed (18), but its importance has not been assessed.

### Target-Site Mechanisms

Alterations of amino acids responsible for insecticide binding at its site of action cause the insecticide to be less effective or even ineffective. The target of organophosphorus (OPs) (e.g., malathion, fenitrothion) and carbamate (e.g., propoxur, sevin) insecticides is acetylcholinesterase in nerve synapses, and the target of organochlorines (DDT) and synthetic pyrethroids are the sodium channels of the nerve sheath. DDT-pyrethroid cross-resistance may be produced by single amino acid changes (one or both of two known sites) in the axonal sodium channel insecticide-binding site (19,20). This cross-resistance appears to produce a shift in the sodium current activation curve and cause low sensitivity to pyrethroids (21). Similarly, cyclodiene (dieldrin) resistance is conferred by single nucleotide changes within the same codon of a gene for a γ-aminobutyric acid (GABA) receptor (22). At least five point mutations in the acetylcholinesterase insecticide-binding site have been identified that singly or in concert cause varying degrees of reduced sensitivity to OPs and carbamate insecticides (23).

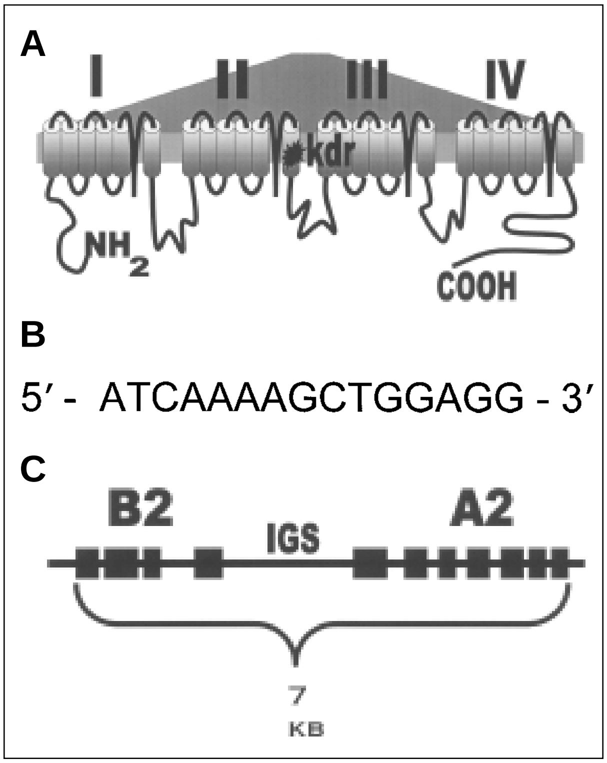

Figure 1. Examples (drawn from references cited in the text) of biochemical resistance mechanisms on the molecular level. A. Single amino acid mutation in the IIS6 membrane-spanning region of the sodium channel gene that confers target-site DDT-pyrethroid resistance in *Anopheles gambiae*. The same mutated codon produces resistance in insects as diverse as mosquitoes, cockroaches, and flies. B. Regulatory element (found upstream of coding sequence) termed the "Barbie Box" that allows induction of insecticide detoxifying oxidase and esterase resistance genes. Many such putative control elements have been found associated with vector resistance enzymes. C. Esterase A2-B2 amplicon. These resistance esterase genes lie 5' end to 5' end within the same amplification unit. More than 100 copies of this amplicon may be present in a single mosquito. This is one example of a family of amplified esterase genes.

### Detoxification Mechanisms

The enzymes responsible for detoxification of xenobiotics in living organisms are transcribed by members of large multigene families of esterases, oxidases, and GST. Perhaps the most common resistance mechanisms in insects are modified levels or activities of esterase detoxification enzymes that metabolize (hydrolyze ester linkages) a wide range of insecticides. These esterases comprise six families of proteins belonging to the α/ß hydrolase fold superfamily (24,25). In Diptera, they occur as a gene cluster on the same chromosome (26,27). Individual members of the gene cluster may be modified in instances of insecticide resistance, for example, by changing a single amino acid that converts the specificity of an esterase to an insecticide hydrolase (28) or by existing as multiple-gene copies that are amplified in resistant insects (the best studied examples are the B1 [29] and A2-B2 [30] amplicons in *Culex pipiens* and *C. quinquefasciatus*).

The cytochrome P450 oxidases (also termed oxygenases) metabolize insecticides through O-, S-, and N-alkyl hydroxylation, aliphatic hydroxylation and epoxidation, aromatic hydroxylation, ester oxidation, and nitrogen and thioether oxidation (31). The cytochrome P450s belong to a vast superfamily. Of the 62 families of P450s recognized in animals and plants, at least four (families 4,6,9,18) have been isolated from insects. The insect P450 oxidases responsible for resistance have belonged to family 6, which, like the esterases, occur in Diptera as a cluster of genes (32). Members of the cluster may be expressed as multiple (up to five) alleles (33). Enhanced levels of oxidases in resistant insects result from constitutive overexpression rather than amplification (34,35). The mechanisms of oxidase overproduction in resistance are under extensive investigation and appear to result from both cis- and trans-acting factors, perhaps associated with the phenomenon of induction (36-38).

Most organisms possess multiple GST from two or more classes (39). GST implicated in DDT insecticide resistance exist as clusters of genes that have been further shuffled through the genome by recombination (40). A number of resistance GST genes, including multiple forms in the same insect, have been characterized in vectors (41-43).

### Resistance to Growth Regulators, Ivermectins, and Other Microbial Agents

Because of their more recent introduction to vector control programs, we discuss growth regulators, ivermectins, and other microbial agents as a group. The initial mechanisms that conferred resistance to insect growth regulators were oxidase-based (44). Resistance to ivermectins has resulted from a number of factors, including oxidase, conjugation, and altered target-site mechanisms (45). Vectors have not yet demonstrated resistance to these compounds in the field.

Microbial agents such as *Bacillus sphaericus* and *B. thuringiensis* are considered insecticides because the principal active agents are crystal toxins produced by the bacteria. The mechanisms of resistance to *B. sphaericus* are not yet defined (46,47), but more than one mechanism seems to be involved (48). Resistance to *B. thuringiensis* has resulted from reduced binding of the toxin to the brush border in the lumen of the insect gut (49,50) or by enhanced digestion of toxin by gut proteases (51). The six different toxin types in the *B. thuringiensis israelensis* strain used for vector control were expected to retard or prevent development of a comprehensive resistance mechanism; however, multitoxin resistance to *B. T. israelensis* has already appeared (52,53).

### Detecting and Monitoring Resistance

The initial step in identifying a potential problem is to detect changes in the susceptibility of a population of vectors—through bioassay, biochemical assay, or molecular assay (Figure 2).

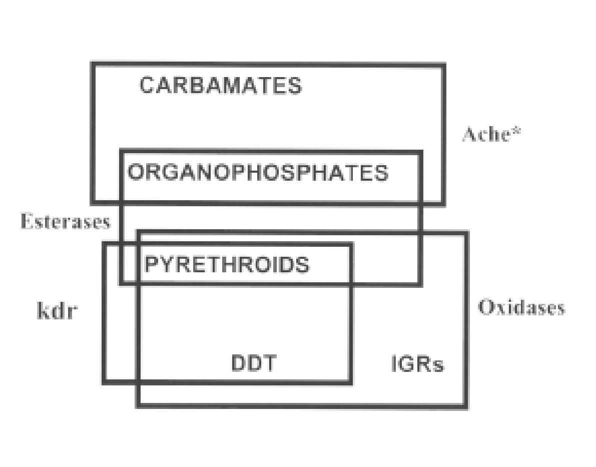

Figure 2. Cross-resistance relationships of commonly used classes of insecticides.

### Bioassays

WHO has developed susceptibility bioassay tests (available in kit form for purchase from WHO) for mosquitoes, lice, bedbugs, reduviid bugs, cockroaches, blackflies, houseflies, ticks, and fleas (15). Our laboratory found that time-mortality bioassays were more sensitive than dose-mortality bioassays in detecting changes in susceptibility and had better correlation with microplate-based biochemical assays for resistance mechanisms (54,55). Time-based bioassays have been further modified through the use of insecticide-coated glass bottles and solutions of standard-grade insecticides or synergists; this approach simplifies the bioassay process and increases the amount of information that can be obtained from a limited pool of mosquitoes (56).

### Biochemical and Molecular Assays

Biochemical and molecular methods can detect resistance mechanisms in individual insects; therefore, they can confirm resistance with the use of only a small number of insects. Identification of resistance mechanisms helps determine the cross-resistance spectrum (Figure 3), facilitates the choice of alternative insecticides, and allows detailed mapping of areas with resistant populations. Specific biochemical assays have been developed for all known resistance mechanisms, except the modified sodium and GABA receptor mechanisms (57-59).

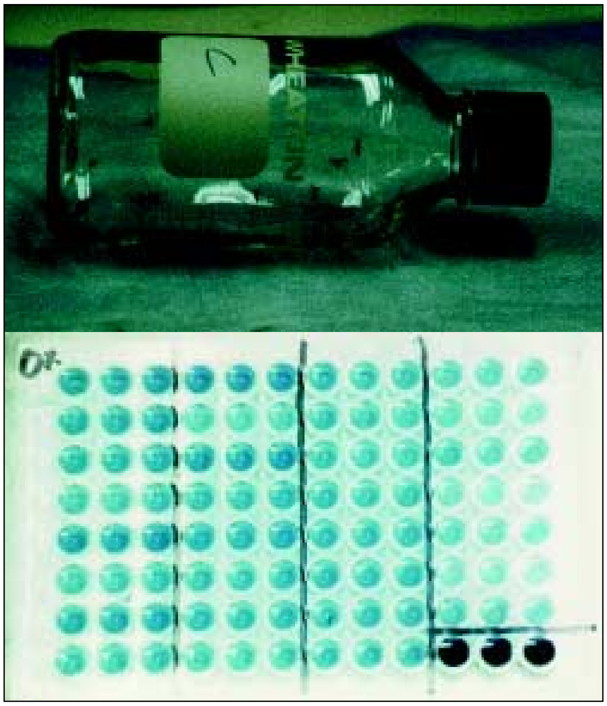

Figure 3. Examples of simple diagnostic assays for insecticide resistance include bioassays run in treated bottles (upper) and biochemical detection and measurement of resistance enzyme activity in microplates (lower).

Molecular information on resistance mechanisms will increasingly be incorporated into resistance diagnostic procedures. One type of mechanism that will become much easier to detect will be the point mutations that cause target-site resistance or changes in detoxification enzyme specificity. Thus far, target-site mechanisms have been detected by polymerase chain reaction-restriction enzyme (PCR-REN) and PCR amplification of specific alleles (60,61).

### Features of Resistance Emergence

Innumerable genetic, biologic, and operational factors influence the development of insecticide resistance. In many respects, resistance is a chaotic problem, with different outcomes possible in a particular area, depending on the influence of diverse factors on initial conditions. Even so, certain factors affect resistance development throughout the world. We discuss major resistance characteristics and show why each manifestation of resistance is potentially unique and therefore must be independently evaluated.

### Focal Nature of Resistance

Vector control personnel frequently assume that resistance in a particular species occurs throughout their control area, but in reality, insecticide resistance is focal. In Guatemala, sampling sites for *Anopheles albimanus* only a few kilometers apart varied not only by presence or absence of resistance, but also by level of resistance and by dominant mechanism responsible for resistance (55).

Surveillance data (Brogdon and McAllister, unpub. data) from the United States show that resistance to OPs in *Culex* mosquito species is focal in a number of states—generally high in urban areas and absent in rural sites. The higher levels of resistance are in areas of ongoing control activities. When resistance levels in adjacent counties were compared, levels were higher in areas of intensive mosquito control.

While the relative importance of agricultural versus public health use of insecticides to resistance development has been widely argued

(62), resistance has been associated with both uses. Agricultural use caused resistance in Central American *An. albimanus* (55). However, in Haiti (54) and in Sudan (62), the impact of public health spraying on development of resistance is clear. In Sri Lanka, resistance in one vector, *An. cuilifacies*, is characteristic of public health spraying, while resistance in another, *An. nigerrimus*, has a profile that indicates agricultural chemicals (62). Resistance appears rapidly in areas where bed nets are used to control mosquitoes (Western Kenya) or sandflies (Colombia) (63; Brogdon and McAllister, unpub. data).

### Resistance and Disease-Endemic Areas

Vector control is affected not only by the focal nature and distribution of resistance but also by disease incidence. Resistance detection could actually interfere with disease control programs if adequate surveillance data are not also collected. In Ecuador (57) we observed OPs resistance in the malaria vector *An. albimanus* in the central agricultural provinces of Guayas, Manabi, and Los Rios, the sources of the country's resistance surveillance data. However, in the northern province of Esmeraldas, where insecticide use had been limited and an epidemic of *Plasmodium falciparum* malaria was under way, populations were completely susceptible. Lack of insecticide use does not preclude immigration of resistance genes (e.g., the movement of esterase resistance to OPs in *C. pipiens* into certain areas of France [64]).

### Resistance and Disease Control

To compromise insecticide vector control, the level of resistance must be high enough to adversely affect disease transmission. In many cases, vector control may not be affected by the level of resistance. For example, an activity may be controlling only 75% of the vector population. If, for example, the level of resistance is lower than 10%, resistance will not affect disease control efforts; in this situation, increasing surveillance and monitoring level and frequency of resistance would be sufficient. No change in control methods would be needed. Western Kenya is a good operational example of the coexistence of resistance and disease control. Pyrethroid resistance appeared soon after bed nets were introduced (64). After 2 years, the resistance level had not changed significantly, possibly because of the continual massive introduction of susceptible genes (65). Other reasons may explain why the presence of insecticide resistance genes in vectors in a control area does not mean that effective control is not being achieved. For example, resistance genes may not be expressed, they may be expressed in an alternative stage of development to that being controlled by insecticide, or the gene detected may be a member of an alternative gene subfamily to one that can affect the compound being used. We have observed in *An. albimanus* and *An. gambiae* that resistance enzymes, especially esterases and GST, may be expressed only in freshly emerged adult anophelines and may be absent in older mosquitoes, those potentially infective for malaria (Brogdon and McAllister, unpub. data).

### Pyrethroid Resistance

Pyrethroid resistance is emerging despite early optimism that because of its rapid toxicologic action this newest large class of insecticides would not produce resistance (66). Resistance is not evolving through unique new mechanisms; rather, existing mechanisms are being enhanced, and cross-resistance is occurring. In Guatemala, pyrethroid resistance was first reported in an *An. albimanus* population resistant to fenitrothion. When deltamethrin was used, the esterase conferring fenitrothion resistance was enhanced by selective pressure to produce deltamethrin cross-resistance (67). Additionally, we are now finding DDT-permethrin cross-resistance due to oxidase cross-resistance in the same mosquito. A similar pattern of cross-resistance has been documented for *C. pipiens* in Ohio. Multiresistance (two or more resistance mechanisms in the same insect) is becoming widespread as control programs make sequential use of one chemical class after another.

A far more threatening development in pyrethroid resistance is the appearance of target-site resistance (also termed knockdown resistance) to pyrethroids in several important vectors in multiple locations. We have detected the knockdown resistance mechanism in the dengue and yellow fever vector *Aedes aegypti* from Puerto Rico and Indonesia and in the encephalitis vector *C. quinquefasciatus* from Louisiana. French researchers have detected the mechanism in *An. gambiae*, the primary African vector of malaria, in several countries of West

Africa (68,69). This resistance mechanism may be a legacy of similarities in the site of action of pyrethroids and DDT.

### Prospects for Resistance Management

A National Research Council report (70) on strategies and tactics for pesticide resistance management described insecticide susceptibility as a resource and resistance surveillance as an essential step in resistance management. Resistance surveillance has three objectives: 1) Provide baseline data for program planning and pesticide selection before the start of control operations; 2) Detect resistance at an early stage so that timely management can be implemented (even detection of resistance at a late stage can be important in elucidating the causes of failure of disease control; however, in such cases, any management other than replacement of the pesticide may not be possible); 3) Continuously monitor the effect of control strategies on resistance.

Resistance to insecticides—even to pyrethroids—in disease vectors is widespread. With the availability of more sensitive and easy-to-use surveillance techniques (56,58,59), the means for managing resistance are at hand. A challenge to resistance management is that efforts to control vector-borne diseases are becoming more diversified; the global effort to control malaria is a case in point. In the 1950s, WHO mounted a global effort to eradicate malaria. Failure of this effort was caused by many factors, including insecticide resistance. Now the best prospect for a worldwide malaria vector control strategy appears to be the use of pyrethroid-impregnated bed nets, because they are less expensive than spraying walls with residual insecticide, are effective in reducing child deaths, and can be better administered through a horizontal, community-based program (71). This type of vector control means that resistance surveillance will also have to be handled and interpreted locally. Decisions will have to be made within local programs by the end user. Moreover, as urbanization increases around the world, some horizontal programs are better able financially to contract out control and surveillance activities. In the United States, local control programs use diverse methods for vector control to meet their specific, often unique needs (72). Diverse methods will likely become the characteristic approach to vector control worldwide, given the prohibitive economics of mounting a worldwide disease control campaign based on vertical programs and the availability of entrepreneurs that increasingly contract for vector control on a city-by-city basis internationally (73-75). In vertical programs, resistance surveillance was a component more in theory than in fact. The challenge will be to maximize the exposure of vector control personnel and entrepreneurs to management principles and to make widely available the surveillance tools required.

### Priorities for the Future

Sufficient means exist to detect and manage resistance at a higher level and with greater effectiveness. A number of training courses and consultations have been conducted to broaden the base of resistance surveillance in the United States. However, the need for resistance surveillance information is global. Perhaps the most cost-effective way to disseminate this information will be the Internet.

Internationally, the increasing diversity of vector control measures will require continued development of simple and informative methods. How can we use the most sensitive and informative molecular and biochemical methods in concert with bioassay techniques? How can these be best simplified so that relatively untrained personnel can use them?

While initial detection and field surveillance for resistance will likely continue to be based upon simple bioassay, biochemical, and molecular tools, the deeper understanding of how resistance arises and maintains itself in populations requires molecular genetics studies.

The most complete understanding of insecticide resistance mechanisms in disease vectors has come from studies with mosquitoes. Much more attention is needed for resistance detection and surveillance methods in other vector groups, such as sand flies, triatomids, lice, fleas, and ticks. Most important, however, is that resistance detection be made an integral part of all control programs. The resources for vector control, even under emergency situations, are limited and must be used as effectively as possible.

Dr. Brogdon is a research entomologist with the National Center for Infectious Diseases, CDC. His research interests include insecticide resistance in disease vectors; vector biochemistry, physiology, and molecular biology; and mosquito behavior.

Dr. McAllister is a visiting fellow funded by the Emerging Infections Program, CDC.

### References

1. Krogstad DJ. Malaria as a reemerging disease. Epidemiol Rev 1996;18:77-89.
2. Roberts DR, Laughlin LL, Hsheih P, Legters LJ. DDT, global strategies, and a malaria control crisis in South America. Emerg Infect Dis 1997;3:295-302.
3. Brandling-Bennett AD, Pinheiro F. Infectious diseases in Latin America and the Caribbean: are they really emerging and increasing? Emerg Infect Dis 1996;2:59-61.
4. Gubler DJ, Clark GG. Dengue/dengue hemorrhagic fever: the emergence of a global health problem. Emerg Infect Dis 1995;1:55-7.
5. Briseno-Garcia B, Gomez-Dantes H, Argott-Ramirez E, Montesano R, Vazquez-Martinez A-L, Ibanez-Bernal S, et al. Potential risk for dengue hemorrhagic fever: the isolation of serotype dengue-3 in Mexico. Emerg Infect Dis 1996;2:133-5.
6. Sanders EJ, Borus P, Ademba G, Kuria G, Tukei PM, LeDuc JW. Sentinel surveillance for yellow fever in Kenya, 1993 to 1995. Emerg Infect Dis 1996;2:236-8.
7. Meslin F-X. Global aspects of emerging and potential zoonoses: a WHO perspective. Emerg Infect Dis 1997;3:223-8.
8. Thompson DF, Malone JB, Harb M, Faris R, Huh OK, Buck AA, et al. Bancroftian filariasis distribution and diurnal temperature differences in the Southern Nile Delta. Emerg Infect Dis 1996;2:234-5.
9. Daniels TJ, Falco RC, Schwartz I, Varde S, Robbins RG. Deer ticks (*Ixodes scapularis*) and the agents of Lyme disease and human granulocytic ehrlichiosis in a New York City park. Emerg Infect Dis 1997;3:353-5.
10. Walker DH, Dumler JS. Emergence of erlichiosis as human health problem. Emerg Infect Dis 1996;2:18-29.
11. Jackson LA, Spach DH. Emergence of *Bartonella quintana* infection among homeless persons. Emerg Infect Dis 1996;2:141-3.
12. Azad AF, Radulovic S, Higgins JA, Noden BH, Troyer JM. Flea-borne rickettsioses: ecologic considerations. Emerg Infect Dis 1997;3:319-27.
13. Raoult D, Roux V, Ndihokubwayo JB, Bise G, Baudon D, Martet G, et al. Jail fever (epidemic typhus) outbreak in Burundi. Emerg Infect Dis 1997;3:357-60.
14. Smoak BL, McClain JB, Brundage JF, Broadhurst L, Kelly DJ, Dasch GA, et al. An outbreak of spotted fever rickettsiosis in U.S. Army troops deployed to Botswana. Emerg Infect Dis 1996;2:217-21.
15. Vector resistance to insecticides. 15th Report of the WHO Expert Committee on Vector Biology and Control. World Health Organ Tech Rep Ser 818 1992;818:1-62.
16. Rodhain F. Donnes recentes sur l'epidemiologie de l'encephalite japonaise. Bull Acad Natl Med 1996;180:1325-37.
17. Centers for Disease Control and Prevention. Public health consequences of a flood disaster—Iowa, 1993. MMWR Morb Mortal Wkly Rep 1993;270:1406-8.
18. Patil NS, Lole KS, Deobagkar DN. Adaptive larval thermotolerance and induced cross-tolerance to propoxur insecticide in mosquitoes *Anopheles stephensi* and *Aedes aegypti*. Med Vet Entomol 1996;10:277-82.
19. Miyazaki M, Ohyama K, Dunlap DY, Matsumura F. Cloning and sequencing of the *para*-type sodium channel gene from susceptible and *kdr*-resistant German cockroaches (*Blatella germanica*) and house fly (*Musca domestica*). Mol Gen Genet 1996;252:61-8.
20. Williamson MS, Martinez-Torrez D, Hick CA, Devonshire AL. Identification of mutations in the housefly *para*-type sodium channel gene associated with knockdown resistance (*kdr*) to pyrethroid insecticides. Mol Gen Genet 1996;252:51-60.
21. Vais H, Williamson MS, Hick CA, Eldursi N, Devonshire AL, Usherwood PN. Functional analysis of a rat sodium channel carrying a mutation for insect knock-down resistance (kdr) to pyrethroids. FEBS Lett 1997;413:327-32.
22. Ffrench-Constant RH, Steichen J, Rocheleau TA, Aronstein K, Roush RT. A single amino acid substitution in a β–aminobutyric acid subtype A receptor locus associated with cyclodiene insecticide resistance in *Drosophila* populations. Proc Natl Acad Sci U S A 1993;90:1957-61.
23. Mutero A, Pralavorio M, Bride JM, Fournier D. Resistance-associated point mutations in insecticide-insensitive acetylcholinesterase. Proc Natl Acad Sci U S A 1994;91:5922-6.
24. Cygler M, Schrag JD, Sussman JL, Harel M, Silman I, Gentry MK, et al. Relationship between sequence conservation and three-dimensional structure in a large family of esterases, lipases and related proteins. Protein Sci 1993;2:366-82.
25. Oakeshott JG, van Papenrecht EA, Boyce TM, Healy MJ, Russell RJ. Evolutionary genetics of *Drosophila* esterases. Genetica 1993;90:239-68.
26. Campbell PM, Trott JF, Claudianos C, Smyth KA, Russell RJ, Oakeshott JG. Biochemistry of esterases associated with organophosphorus resistance in *Lucilia cuprina* with comparisons to putative orthologues in other Diptera. Biochem Genet 1997;35:17-40.
27. Russell RJ, Robin GC, Kostakos P, Newcomb RD, Boyce TM, Medveczky KM, et al. Molecular cloning of an α-esterase gene cluster on chromosome 3R of *Drosophila melanogaster*. Insect Biochem Mol Biol 1996;26:235-47.
28. Newcomb RD, Campbell PM, Ollis DL, Cheah E, Russell RJ, Oakeshott JG. A single amino acid substitution converts a carboxylesterase to an organophosphorus hydrolase and confers insecticide resistance on a blowfly. Proc Natl Acad Sci U S A 1997;94:7464-8.
29. Mouches C, Pauplin Y, Agarwal M, Lemieux L, Herzog M, Abadon M, et al. Characterization of amplification core and esterase B1 gene responsible for insecticide resistance in *Culex*. Proc Natl Acad Sci U S A 1990;87:2574-8.
30. Vaughan A, Hawkes N, Hemingway J. Co-amplification explains linkage disequilibrium of two mosquito esterase genes in insecticide-resistant *Culex quinquefasciatus*. Biochem J 1997;325:359-65.
31. Wilkinson CF. Insecticide biochemistry and physiology. New York: Plenum Press; 1976. p. 768.
32. Maitra S, Dombroski SM, Waters LC, Ganguly R. Three second chromosome-linked clustered Cyp6 genes show differential constitutive and barbital-induced expression in DDT-resistant and susceptible strains of *Drosophila melanogaster*. Gene 1996;180:165-71.

33. Tomita T, Liu N, Smith FF, Sridhar P, Scott JG. Molecular mechanisms involved in increased expression of a cytochrome P450 responsible for pyrethroid resistance in the housefly, *Musca domestica*. Insect Mol Biol 1995;4:135-40.
34. Tomita T, Scott JG. cDNA and deduced protein sequence of Cyp6D1: the putative gene for a cytochrome P450 responsible for pyrethroid resistance in house fly. Insect Biochem Mol Biol 1995;25:275-83.
35. Carino FA, Koener JF, Plapp FW Jr, Feyereisen R. Constitutive overexpression of the cytochrome P450 gene Cyp6A1 in a house fly strain with metabolic resistance to insecticides. Insect Biochem Mol Biol 1994;24:411-8.
36. Cohen MB, Koener JF, Feyereisen R. Structure and chromosomal localization of Cyp6A1, a cytochrome P450-encoding gene from the house fly. Gene 1994;146:267-72.
37. Brun A, Cuany A, LeMouel T, Berge J, Amichot M. Inducibility of the *Drosophila melanogaster* cytochrome P450 gene, Cyp6A2, by phenobarbital in insecticide susceptible or resistant strains. Insect Biochem Mol Biol 1996;26:697-703.
38. Liu N, Scott JG. Phenobarbital induction of Cyp6D1 is due to a *trans* acting factor on autosome 2 in house flies, *Musca domestica*. Insect Mol Biol 1997;6:77-81.
39. Hayes JD, Pulford DJ. The glutathione S-transferase supergene family: regulation of GST and the contribution of the isoenzymes to cancer chemoprotection and drug resistance. Crit Rev Biochem Mol Biol 1995;30:445-600.
40. Zhou Z-H, Syvanen M. A complex glutathione transferase gene family in the housefly *Musca domestica*. Mol Gen Genet 1997;256:187-94.
41. Grant DF, Dietze EC, Hammock BD. Glutathione S-transferase isozymes in *Aedes aegypti* purification, characterization, and isozyme-specific regulation. Insect Biochem 1991;21:421-33.
42. Prapanthadara L, Koottathep S, Promtet N, Hemingway J, Ketterman AJ. Purification and characterization of a major glutathione S-transferase from the mosquito *Anopheles dirus* (species B). Insect Biochem Mol Biol 1996;26:277-85.
43. Ranson H, Prapanthadara L, Hemingway J. Cloning and characterization of two glutathione S-transferases from a DDT-resistant strain of *Anopheles gambiae*. Biochem J 1997;324:97-102.
44. Kotze AC, Sales N, Barchia IM. Diflubenzuron tolerance associated with monooxygenase activity in field strain larvae of the Australian sheep blowfly (Diptera: Calliphoridae). J Econ Entomol 1997;90:15-20.
45. Clark JM, Scott JG, Campos F, Bloomquist JR. Resistance to ivermectins: extent, mechanisms, and management. Annu Rev Entomol 1995;40:1-30.
46. Rao DR, Mani TR, Rajendran R, Joseph AS, Gajanana A, Reuben R. Development of a high level of resistance to *Bacillus sphaericus* in a field population of *Culex quinquefasciatus* from Kochi, India. Journal of the American Mosquito Control Association 1995;11:1-5.
47. Rodcharoen J, Mulla MS. Cross-resistance to *Bacillus sphaericus* strains in *Culex quinquefasciatus*. J Am Mosq Control Assoc 1996;12:247-50.
48. Nielsen-Leroux C, Pasquier F, Charles JF, Sinegre G, Gaven B, Pasteur N. Resistance to *Bacillus sphaericus* involves different mechanisms in *Culex pipiens* (Diptera:Culicidae) larvae. J Med Entomol 1997;34:321-7.
49. Escriche B, Tabashnik B, Finson N, Ferre J. Immunohistochemical detection of binding of CryIA crystal proteins of *Bacillus thuringiensis* in highly resistant strains of *Plutella xylostella* (L.) From Hawaii. Biochem Biophys Res Commun 1995;212:388-95.
50. Tabashnik BE, Malvar T, Liu YB, Finson N, Borthakur D, Shin BS, et al. Cross-resistance of the diamondback moth indicates altered interactions with domain II of *Bacillus thuringiensis* toxins. Appl Environ Microbiol 1996;62:2839-44.
51. Keller M, Sneh B, Strizhov N, Prudovsky E, Regev A, Koncz C, et al. Digestion of delta-endotoxin by gut proteases may explain reduced sensitivity of advanced instar larvae of *Spodoptera littoralis* to CryIC. Insect Biochem Mol Biol 1996;26:365-73.
52. Tabashnik BE, Liu YB, Finson N, Masson L, Heckel DG. One gene in diamondback moth confers resistance to four *Bacillus thuringiensis* toxins. Proc Natl Acad Sci U S A 1997;94:1640-4.
53. Cheong H, Dhesi RK, Gill SS. Marginal cross-resistance to mosquitocidal *Bacillus thuringiensis* strains in Cry11A-resistant larvae: presence of Cry11A-like toxins in these strains. FEMS Microbiol Lett 1997;153:419-24.
54. Brogdon WG, Hobbs JH, St. Jean Y, Jacques JR, Charles LB. Microplate assay analysis of reduced fenitrothion susceptibility in Haitian *Anopheles albimanus*. J Am Mosq Control Assoc 1988a;4:152-8.
55. Brogdon WG, Beach RF, Stewart JM, Castanaza L. Microplate assay analysis of the distribution of organophosphate and carbamate resistance in Guatemalan *Anopheles albimanus*. Bull World Health Organ 1988b;66:339-46.
56. Brogdon WG, McAllister JC. Simplification of adult mosquito bioassays through use of time-mortality determinations in bottles. J Am Mosq Control Assoc. In press 1998.
57. Brogdon WG. Biochemical resistance detection: an alternative to bioassay. Parasitology Today 1989;5:56-60.
58. Brogdon WG, Barber AM. Microplate assay of glutathione S-transferase activity for resistance detection in single-mosquito homogenates. Comp Biochem Physiol 1990;96B:339-42.
59. Brogdon WG, McAllister JC. Heme peroxidase activity measured in single mosquitoes identifies individuals expressing an elevated oxidase for insecticide resistance. J Am Mosq Control Assoc 1997;13:233-7.
60. Field LM, Crick SE, Devonshire AL. Polymerase chain reaction-based identification of insecticide resistance genes and DNA methylation in the aphid *Myzus persicae* (Sulzer). Insect Mol Biol 1996;5:197-202.
61. Steichen JC, Ffrench-Constant RH. Amplification of specific cyclodiene insecticide resistance alleles by the polymerase chain reaction. Pesticide Biochemistry and Physiology 1994;48:1-7.
62. Lines JD. Do agricultural insecticides select for insecticide resistance in mosquitoes? A look at the evidence. Parasitology Today 1988;4:S17-S20.

63. Vulule JM, Beach RF, Atieli FK, Roberts JM, Mount DL, Mwangi RW. Reduced susceptibility of *Anopheles gambiae* to permethrin associated with the use of permethrin-impregnated bednets and curtains in Kenya. Med Vet Entomol 1994;8:71-5.
64. Rivet Y, Raymond M, Rioux JA, Delalbre A, Pasteur N. Resistance monitoring in *Culex pipiens* (Diptera:Culicidae) from central-eastern France. J Med Entomol 1994;31:231-9
65. Vulule JM, Beach RF, Atieli FK, Mount DL, Roberts JM, Mwangi RW. Long-term use of permethrin-impregnated nets does not increase *Anopheles gambiae* permethrin tolerance. Med Vet Entomol 1996;10:71-9.
66. Malcolm CA. Current status of pyrethroid resistance in anophelines. Parasitology Today 1988;4:S13-S6.
67. Brogdon WG, Barber AM. Fenitrothion-deltamethrin cross-resistance conferred by esterases in Guatemalan *Anopheles albimanus*. Pesticide Biochemistry and Physiology 1990;37:130-9.
68. Elissa N, Mouchet J, Riviere F, Meunier JY, Yao K. Resistance of *Anopheles gambiae* s.s. to pyrethroids in Cote d'Ivoire. Ann Belge Med Trop 1993;73:291-4.
69. Martinez-Torres D, Chandre F, Williamson MS, Darriet F, Berge JB, Devonshire AL, et al. Molecular characterization of pyrethroid resistance in the malaria vector *Anopheles gambiae*. Insect Molecular Science. In press, 1998.
70. National Research Council. Strategies and tactics for management. In: Pesticide resistance. Washington: The Academy; 1986. p. 471.
71. Lengeler C, Snow RW. From efficacy to effectiveness: insecticide-treated bednets in Africa. Bull World Health Organ 1996;74:325-32.
72. Challet GL. Elements of a vector control program. J Am Mosq Control Assoc 1991;7:103-6.
73. Vector control for malaria and other mosquito-borne diseases. Report of a WHO study group. World Health Organ Tech Rep Ser 1995;857:1-91.
74. Gratz NG, Jany WC. What role for insecticides in vector control programs? Am J Trop Med Hyg 1994;50:11-20.
75. Kanda T, Bunnag D, Deesin V, Deesin T, Leemingsawat S, Komalamisra N, et al. Integration of control measures for malaria vectors in endemic areas of Thailand. Southeast Asian J Trop Med Public Health 1995;26:154-63.
